# Green and Roasted Coffee Extracts Inhibit Interferon-β Release in LPS-Stimulated Human Macrophages

**DOI:** 10.3389/fphar.2022.806010

**Published:** 2022-05-05

**Authors:** Valentina Artusa, Carlotta Ciaramelli, Alessia D’Aloia, Fabio Alessandro Facchini, Nicole Gotri, Antonino Bruno, Barbara Costa, Alessandro Palmioli, Cristina Airoldi, Francesco Peri

**Affiliations:** ^1^ Dipartimento di Biotecnologie e Bioscienze, Università Degli Studi di Milano-Bicocca, Milano, Italy; ^2^ Milan Center for Neurosciences, Università Degli Studi di Milano-Bicocca, Milano, Italy; ^3^ Laboratory of Immunology and General Pathology, Department of Biotechnologies and Life Science, University of Insubria, Varese, Italy; ^4^ Laboratory of Innate Immunity, IRCCS MultiMedica, Polo Scientifico e Tecnologico, Milano, Italy

**Keywords:** coffee extracts, chlorogenic acid, 5-CQA, macrophages, inflammation, immunomodulation, interferon-β

## Abstract

The anti-inflammatory activity of coffee extracts is widely recognized and supported by experimental evidence, in both *in vitro* and *in vivo* settings, mainly murine models. Here, we investigated the immunomodulatory properties of coffee extracts from green (GCE) and medium-roasted (RCE) *Coffea canephora* beans in human macrophages. The biological effect of GCE and RCE was characterized in LPS-stimulated THP-1-derived human macrophages (TDM) as a model of inflammation. Results showed decreased amounts of TNF-α, IL-6 and IL-1β and a strong dose-dependent inhibition of interferon-β (IFN-β) release. Molecular mechanism of IFN-β inhibition was further investigated by immunofluorescence confocal microscopy analysis that showed a diminished nuclear translocation of p-IRF-3, the main transcription factor responsible for IFN-β synthesis. The inhibition of IFN-β release by RCE and GCE was also confirmed in human primary CD14^+^ monocytes-derived macrophages (MDM). The main component of coffee extracts, 5-*O*-caffeoylquinic acid (5-CQA) also inhibited IFN-β production, through a mechanism occurring downstream to TLR4. Inhibition of IFN-β release by coffee extracts parallels with the activity of their main phytochemical component, 5-CQA, thus suggesting that this compound is the main responsible for the immunomodulatory effect observed. The application of 5-CQA and coffee derived-phytoextracts to target interferonopathies and inflammation-related diseases could open new pharmacological and nutritional perspectives.

## Introduction

Chronic inflammation is largely recognized as a relevant hallmark in diverse pathologies, ranging from allergic reactions to autoimmune, infectious, cardiovascular diseases, atherosclerosis, and cancer (Chen et al., 2018). In this scenario, it is now clear that incorrect lifestyle plays a major role in supporting inflammation, already at the early onset of these disorders (Monteiro and Azevedo 2010; Magnuson et al., 2015; Guha and Majumder 2020). Based on this knowledge, also confirmed in experimental preclinical models and in several clinical settings, strong efforts are currently addressed to the identification and investigation of the molecular mechanisms governing anti-inflammatory and immunomodulatory properties of food-derived natural products/extracts. Bioactive foods rich in anti-inflammatory molecules have been proposed for the prevention and the treatment of a wide spectrum of inflammatory diseases (Watson and Preedy 2019). Lipopolysaccharide (LPS), the major component of Gram-negative bacteria cell wall, is the main pro-inflammatory stimulus that triggers its specific receptor, Toll-like receptor 4 (TLR4) (Raetz and Whitfield 2002), thus inducing the inflammatory response, *via* innate immunity activation(Jerala 2007; Gioannini and Weiss 2007; Ryu et al., 2017). Endogenous danger-associated molecular patterns (DAMPs), such as High Mobility Group Box 1 (HMGB1) and Heat Shock Proteins (HSPs), released from dying or lytic cells during host tissue injury, or viral infection, exacerbate the stimulation of TLR4, thus generating acute or chronic sterile inflammations (Turner 2016; Cruz et al., 2018; Land 2020). TLR4 stimulation induces activation of the MyD88-dependent signaling, leading to the production of inflammatory cytokines and chemokines, mainly tumor necrosis factor-alpha (TNF-α), interleukin 6 (IL-6), interleukin 1-beta (IL-1β) (Lu et al., 2008). As a consequence of CD14-triggered endosomal internalization TLR4 can activate a second pathway, known as the TRIF-dependent pathway, inducing type I IFN production (in particular IFN-β) (Honda et al., 2006; Zanoni et al., 2011). Type I interferons (IFN-I) are antiviral cytokines with immunomodulatory and anti-proliferative function, they downregulate the expression of pro-inflammatory cytokines and MHC class II molecules in antigen presenting cells, while upregulate the expression of anti-inflammatory cytokines, such as IL-10. However, there is evidence of detrimental effects of excessive type I IFN response, in viral and bacterial infections as well as in autoinflammatory syndromes (Davidson et al., 2015). Type I IFNs have been shown to have negative effects in infections with intracellular bacteria such as *Mycobacterium tuberculosis* (Stanley et al., 2007). Moreover, bacterial superinfections following viral infections represent a clear example of a scenario where type I IFNs can misdirect the immune response (Davidson et al., 2015). Inhibition of type-I IFN signaling can therefore have important therapeutic applications as in the case of severe sepsis (Dejager et al., 2014). IFN-I signaling inhibitors and monoclonal antibodies against IFN-α and IFN receptor antagonists have been developed for clinical purposes. AstraZeneca-Medimmune developed anifrolumab (formerly known as MEDI-546), a fully human immunoglobulin G1κ monoclonal antibody directed against IFNAR1 underwent phase III clinical trials for the treatment of systemic *lupus erythematosus* (Chasset and Arnaud 2018). While the anti-inflammatory properties of natural compounds are well documented, modulation of interferon release by phytochemicals present in food and beverages and natural extracts still represent a relevant topic to be explored.

Coffee consumption is associated with various long-term health benefits, in particular on age-associated diseases whose onset is related to chronic inflammation, such as cardiovascular diseases (Rodríguez-Artalejo and López-García 2018), chronic liver diseases (Ruhl and Everhart 2005), diabetes (Kempf et al., 2010), and cancer (Pauwels and Volterrani 2021). The anti-inflammatory effects of coffee were extensively reviewed (Wang and Ho 2009). Coffee contains a number of compounds including caffeine, diterpenes, such as kahweol, and several polyphenols, such as chlorogenic acids (CGAs), which are a large family of (-)-quinic acid esters of hydroxycinnamic acids (mainly caffeic acid, ferulic acid and p-coumaric acid), the most abundant being 5-*O*-caffeoylquinic acid (5-CQA) (Perrone et al., 2008; Clifford et al., 2017).

Kahweol (Kim et al., 2004) and CGAs (Kim et al., 2017) were reported to possess anti-inflammatory effects in lipopolysaccharide (LPS)-activated RAW 264.7 macrophages. Green coffee beans can contain as much as 5–14% of dry weight CGAs, and coffee is the major source in the human diet (Olthof et al., 2001; Farah and Donangelo 2006). Their antioxidant and anti-aging properties were recently reported also in *vivo* in the model organism *Caenorhabditis elegans*, whose lifespan was extended by treatment with green coffee extracts (GCEs) (Amigoni et al., 2017). During the roasting process, and depending on the roasting conditions, natural coffee polyphenols are partially decomposed or bound to polymer structures (e.g. high-molecular weight melanoidins), and CGA concentration decreases(Moon et al., 2009; Wei et al., 2012b; Moreira et al., 2012). In murine models of LPS-induced inflammation, light and medium degree roasted coffee reduced IL-6 cytokine expression in LPS treated animals by inhibiting the NF-κB pathway, and this effect decreased by increasing roasting degree (Choi et al., 2018). Interestingly, high- and low-molecular weight melanoidins and small molecules, as pyrocatechol deriving respectively from the Maillard reaction and from thermal degradation during the roasting process, showed to possess anti-inflammatory activity. The studies related to the anti-inflammatory action of CGAs in green coffee extracts and/or melanoidins and pyrocatechol in roasted coffee extract suggest that the mechanism of action is related to the inhibition of TLR4 intracellular signaling (Ye et al., 2017; Tores de la Cruz et al., 2019; Funakoshi-Tago et al., 2020). However, the majority of available studies on the anti-inflammatory effect of roasting-derived coffee components are based on macrophages of murine origin (such as RAW264.7 cells) and *in vivo* models, suggesting a gap of knowledge in the human settings to be filled in.

Here, we provided an NMR-based metabolic profiling of green and roasted coffee extracts (GCE and RCE) and investigated their modulatory activities on the production of both type I interferons and inflammatory cytokines in human immortalized monocytes-derived macrophages. We also reported confocal microscopy data regarding the intracellular effect of coffee extracts. Besides, we assessed the putative contribution of 5-CQA to the biological activity of coffee extracts. Moreover, we investigated the involvement of TLR4 in the mechanism of action of 5-CQA. Lastly, we assessed the bioactivity of coffee extract on human primary monocytes-derived macrophages.

## Material and Methods

### Green and Roasted Coffee Beans Extraction

Coffee extracts from green (GCE) and medium-roasted (RCE) *Coffea canephora* (Pierre ex Froehner, 1897) beans (var. Robusta, origin Brazil) were obtained using a hydro-alcoholic extraction procedure described in a previous work (Ciaramelli et al., 2018). Ground coffee beans were received from Beyers Koffie, Belgium. Each sample was analyzed in triplicate. Briefly, 200 mg of ground green or roasted coffee beans were extracted with 20 ml of a mixture of acidified (with 0.1 M HCl) water (pH 4.5; 70%) and methanol (30%) by sonication at 37 kHz for 15 min at 30°C in an ultrasound bath (Elmasonic P 30 H, Elma Schmidbauer GmbH, Singen, Germany). After 1 h, solutions were filtered through cotton wool and 0.45 µm PTFE filters (Pall Corporation, Port Washington, NY, United States), concentrated under reduced pressure at 40°C and freeze-dried. Extraction yield (mass of extract/mass of dry matter) has been calculated for each sample and resulted in 20 ± 3.8% in the case of GCE and 26 ± 3.6% in the case of RCE. The lyophilized samples were stored at −20°C (Amigoni et al., 2017).

### NMR-Based Metabolic Profiling

Freeze-dried samples were dissolved in 10 mM deuterated phosphate buffer (PB, pH 7.4) at a final concentration of 5 mg/ml, sonicated (37 kHz, 20 min, Elmasonic P 30 H, Elma Schmidbauer GmbH, Singen, Germany) and centrifuged (9425 × *g*, 10 min, 20°C, ScanSpeed 1730R Labogene, Lynge, Sweden). 4,4-Dimethyl-4-silapentane-1-sulfonic acid (DSS, final concentration 1 mM) was added to the supernatant as an internal reference for concentrations and chemical shift. The pH of each sample was verified with a microelectrode (Mettler Toledo, Columbus, OH, United States) for 5 mm NMR tubes and adjusted to 7.4 with NaOD or DCl. All pH values were corrected for the isotope effect. The acquisition temperature was 25°C. All spectra were acquired on an AVANCE III 600 MHz NMR spectrometer (Bruker, Billerica, MA, United States) equipped with a QCI (^1^H, ^13^C, ^15^N/^31^P and ^2^H lock) cryogenic probe. ^1^H-NMR spectra were recorded with water suppression (cpmgpr1d pulse sequences in Bruker library) and 64 scans, spectral width 20 ppm, relaxation delay 30 s. They were processed with 0.3 Hz line broadening, automatically phased and baseline corrected. Chemical shifts were internally calibrated to the DSS peak at 0.0 ppm. Compound identification and assignment were carried out with the support of 2D NMR experiments, comparison with reported assignments (Wei et al., 2012a) and the SMA (Simple Mixture Analysis) analysis tool integrated in MestreNova Software. The ^1^H,^1^H-TOCSY (Total Correlation SpectroscopY) spectra were acquired with 48 scans and 512 increments, a mixing time of 80 ms and relaxation delay 2 s ^1^H,^13^C-HSQC (Heteronuclear Single Quantum Coherence) spectra were acquired with 48 scans and 256 increments, relaxation delay 2 s.

For coffee bean metabolites quantification, the global spectrum deconvolution (GSD) algorithm, available in the Mnova software package (MestReNova v 14.2.0, 2021, Mestrelab Research, Santiago de Compostela, Spain) was used. Overlapping regions were deconvoluted, and absolute quantification was assessed also for coffee bean metabolites with resonances in rare crowded spectral areas. For each compound, the mean of the different assigned signals was determined.

### Chemicals

3-(4,5-Dimethylthiazol-2-Yl)-2,5-Diphenyltetrazolium Bromide (MTT) and lipopolysaccharide (LPS) from *Escherichia coli* 055:B5 were purchased from Sigma-Aldrich (Sigma-Aldrich, Inc., Saint Louis, MO, United States). Reagent-grade water used to prepare all solutions was obtained from a Milli-Q purification system (Millipore, Bedford, MA, United States).

### Cell Cultures

THP-1 cells (ATCC^®^ TIB-202^™^) have been purchased from ATCC® (Manassas, VA 20110 United States), while THP1-XBlue^™^ cells have been purchased from InvivoGen (InvivoGen, San Diego, CA, United States). Both cell lines were cultured in RPMI 1640, 2 mM L-glutamine, 10% heat-inactivated fetal bovine serum, 100 U/ml-100 μg/ml Pen-Strep. Cells were maintained in a humidified 37°C, 5% CO_2_ incubator. Cells were subcultured every 2 days. Exponentially growing cells were adjusted to 0.5 × 10^6^/ml according to the routine procedure. To maintain selection pressure, 100 μg/ml of Zeocin^™^ (InvivoGen, San Diego, CA, United States) were added to the growth medium of THP1-XBlue^™^ cells every other passage. Media and supplements were purchased from Euroclone (Euroclone S.p.A., Pero, Milan, Italy) unless otherwise stated.

### Generation of THP-1-Derived Macrophages from THP-1 Monocytes

THP-1-derived macrophages (TDM) were generated from THP-1 or THP1-XBlue^™^ monocytic cells (3.5 × 10^5^ cells/ml) by exposure to 25 ng/ml phorbol 12-myristate-13-acetate (PMA) (InvivoGen, San Diego, CA, United States). Aliquots (180 μl/well) of the cell suspensions were loaded into a 96-well plate before culture at 37°C, 5% CO_2_. Following 72 h of differentiation, medium was removed and replaced with fresh medium without PMA prior to further treatments.

### Primary CD14^+^ Monocytes Isolation

Human CD14^+^ monocytes were isolated from total peripheral blood mononuclear cells of healthy donors, by immunomagnetic cell sorting. Samples from healthy donor subjects were obtained within a clinical protocol (n°463-2021), approved by the institutional Ethical Committee at IRCCS MultiMedica Milano, Milan, Italy, in accordance with the Helsinki Declaration of 1975 as revised in 2013. 30 ml of whole blood (<1 h old), collected into EDTA tubes, were subjected to Ficoll Histopaque^®^-1077 (Sigma-Aldrich, Inc., Saint Louis, MO, United States) density gradient stratification at 800 × *g*, for 25 min, room temperature (RT). The white ring interface, enriched in mononuclear cells, was collected and washed in PBS at 300 × *g* for 5 min, RT. Residual red blood cells were removed by treating the cell pellets with 1X Red blood lysis buffer solution (10X stock solution: NH_4_Cl 83g, KHCO_3_ 10 g, EDTA 1 mM, pH 7.2–7.4 in 1 L of H_2_O), incubated for 10 min at 4°C and then washed in PBS at 300 × *g* for 5 min, RT. Monocytes were purified from PBMCs by immunomagnetic cell sorting (positive selection) using CD14 MicroBeads UltraPure (Miltenyi Biotec, Auburn, CA, United States) according to the manufacturer’s instructions.

### Generation of CD14^+^ Monocyte-Derived Macrophages (MDM)

Purified CD14^+^ monocytes (3.5 × 10^5^ cells/ml) were differentiated into adherent monocyte-derived macrophages (MDM), by exposure to 50 ng/ml M-CSF (Miltenyi Biotec, Auburn, CA, United States), in 96-well plates for 7 days, at 37°C, 5% CO_2_. Aliquots (180 μl/well) of the cell suspensions were loaded into a 96-well plate before culture at 37°C, 5% CO_2_. Cells were pulsed with M-CSF every 3 days. Following macrophage differentiation, medium was removed and replaced with fresh RPMI medium, prior to further treatments.

### Cell Viability Assay

TDM and MDM were treated with increasing concentrations of GCE or RCE (10–500 μg/ml). After 24 h, cell supernatants were removed and cell viability was assessed by MTT assay, according to the method first described by Tim Mosmann in 1983 (Mosmann 1983). This colorimetric assay uses reduction of a yellow tetrazolium salt (3-(4,5-dimethylthiazol-2-yl)-2,5-diphenyltetrazolium bromide, or MTT) to measure cellular metabolic activity as an indicator of cell viability. Viable cells contain NAD(P)H-dependent oxidoreductase enzymes which reduce the MTT reagent to formazan, an insoluble crystalline product with a deep purple color. After 4 h-incubation with the MTT solution at 37°C, formazan crystals are then dissolved using a solubilizing solution and absorbance is measured at 570 nm using a plate-reader (LabTech Microplate Reader LT-4000).

### Detection of NF-κB Activation (SEAP Assay)

THP1-XBlue^™^ cells were specifically designed for monitoring the NF-κB signal transduction pathway. THP1-XBlue^™^ were derived from the human THP-1 monocytic cell line by stable integration of an NF-κB-inducible secreted embryonic alkaline phosphatase (SEAP) reporter construct. THP1-XBlue^™^ cells are highly responsive to PRR agonists that trigger the NF-κB pathway. TDM obtained from THP1-XBlue^™^ monocytic cells have been pre-treated with increasing concentration of GCE or RCE (1 h) and then stimulated with 100 ng/ml LPS. In general, cell supernatants were collected after 18 h. Monitoring of NF-κB activation by determining the activity of SEAP in the cell culture supernatant, was assessed with QUANTI-Blue^™^ reagent according to the manufacturer’s instruction (InvivoGen, San Diego, CA, United States). Briefly, 50 µl of the supernatants of SEAP-expressing cells were incubated with 180 µl of QUANTI-Blue^™^ substrate in a 96-well plate for 0.5–4 h at 37°C, then optical density (OD) was measured at 630 nm.

### Enzyme-Linked Immunosorbent Assay (ELISA)

The evaluation of the immunomodulatory activity of coffee extracts was performed by measurement of cytokines and interferons released by human macrophage-like cells. TDM or MDM were pre-treated with fresh RPMI medium containing coffee extracts or 5-CQA (1 h), then cells were stimulated with 100 ng/ml LPS. At defined time points of incubation, cell culture supernatants were collected and stored at −20°C. Samples were analyzed in duplicate in each experiment and at least three experiments were performed. The concentrations of IL-1β, IL-6, TNF-α, IFN-β were detected using commercial ELISA kits according to manufacturer’s instructions (DuoSet^®^ ELISA Development Systems, R&D Systems, Inc., Minneapolis, MN, Canada, United States). Briefly, 100 μl of standards and samples were added in respective wells of 96 wells antibody coated plate and incubate for 2 h. After incubation and washing steps, conjugated secondary antibodies were added for 2 h followed by same washing steps. Then substrate solution was added in each well followed by addition of stop solution. A standard curve was obtained by using two-fold dilutions of the standard for each independent experiment. The concentration of cytokines and interferons was calculated using a standard curve.

### Immunofluorescence Analysis

Cellular localization of the phosphorylated form of the transcription factor IRF-3 (p-IRF-3) was examined by confocal immunofluorescence analysis. TDM (2 × 10^4^ cells/well) were seeded and differentiated into CellCarrier-96 Ultra Microplates (6055500, PerkinElmer Inc., Milan, Italy). After differentiation, cell culture media have been replaced with either fresh RPMI or RPMI supplemented with 250 μg/ml GCE or RCE (1 h pre-treatment ), then cells have been exposed to LPS (2 h). Paraformaldehyde 4% (F8775, Sigma-Aldrich, Inc., Saint Louis, MO 63103, United States) fixed cells were permeabilized with ice-cold 100% methanol, blocked in 1X PBS/5% BSA/0.3% Triton X-100, and labeled with Phospho-IRF-3 (Ser386) (E7J8G) XP® Rabbit mAb (E7J8G, Cell Signaling Technology, Inc.). Cells were then tagged with PhenoVue^™^ Fluor 568 conjugated anti-rabbit secondary antibody (2GXRB568C1, PerkinElmer Inc., Milan, Italy). The nucleus was counter-labeled with PhenoVue^™^ Hoechst 33342 Nuclear Stain (CP71, PerkinElmer Inc., Milan, Italy). Images have been acquired using the Operetta CLS^™^ High-Content Analysis System and analyzed through Harmony 4.5 software (PerkinElmer Inc., Milan, Italy).

### Statistical Analysis

Data related to biological assays were analyzed using GraphPad Prism software (ver.9.0.2, GraphPad Software Inc., San Diego, CA, United States) and the results were shown as means ± standard error of the mean (SEM). Data obtained in three or more independent experiments were compared by one-way analysis of variance (ANOVA) followed by *post hoc* Dunnett’s test. Differences between samples were considered statistically significant when *p* < 0.05. Two groups of data were compared using Student’s t-test and comparison visualized as bars placed above columns (when statistically different, *p* < 0.05).

## Results

### NMR-Based Metabolic Profiling of Coffee Extracts

**FIGURE 1:**
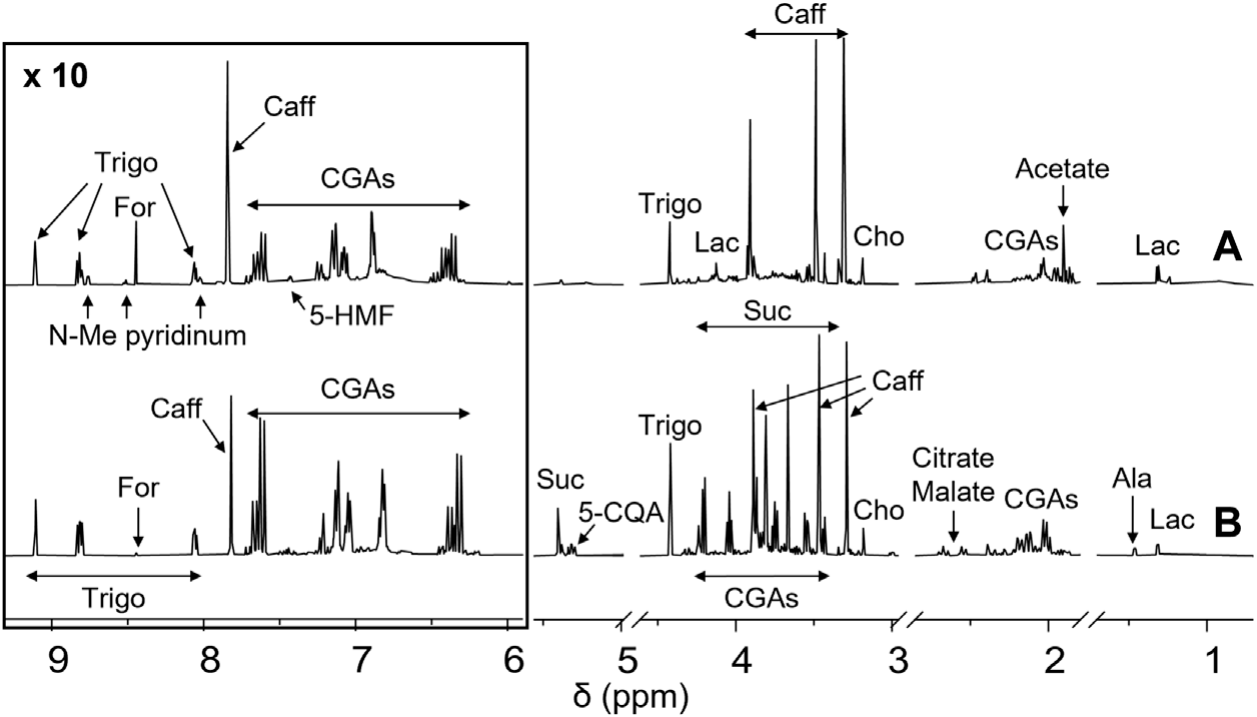
NMR profiling of coffee samples. ^1^H-NMR spectra of Robusta green **(A**, bottom**)** and roasted **(B**, up**)** coffee extracts from Brazil. Each sample, containing 5 mg/ml of extract, was dissolved in a 10 mM deuterated phosphate buffer, pH 7.4, DSS 1 mM. Spectra were acquired at 25°C and 600 MHz. Assignments of the resonances of the most important metabolites are shown (5-HMF, 5-hydroxymethylfurfural; Trigo, trigonelline; N-Me pyridinium, N-methyl pyridinium; For, formate; Caff, caffeine; CGAs, chlorogenic acids; Suc, sucrose; 5-CQA, 5-*O*-caffeoylquinic acid; Cho, choline; Ala, alanine; Lac, lactate).

Green (GCE) and roasted (RCE) coffee beans hydroalcoholic extracts were prepared as previously reported (Amigoni et al., 2017; Palmioli et al., 2017; Ciaramelli et al., 2018; Ciaramelli et al., 2019). The methodology is described in the Material and Methods section. They were characterized by NMR spectroscopy. ^1^H-NMR metabolic profiles of GCE (A) and RCE (B) are depicted in Figure 1.

As previously reported (Ciaramelli et al., 2018; Ciaramelli et al., 2019), the main differences among GCE and RCE rely in the complete disappearance of sucrose in RCE, together with a significant reduction of the amount of CGAs, due to melanoidin formation occurring during the roasting process, and the formation of *N*-methyl pyridinium, nicotinic acid, 5-hydroxy-methyl furfural and 2-furylmethanol. Moreover, a significant decrease in trigonelline and choline content can be observed. Metabolites identification was based on the analysis of mono (^1^H) and bi-dimensional (^1^H,^1^H-TOCSY, ^1^H,^13^C-HSQC) NMR spectra and by the use of specific libraries built in-house for the Simple Mixture Analysis (SMA) tool implemented in the MestReNova 14.2.0 software (mestrelab 2022; Airoldi 2018). Data were in agreement with those previously reported by our and other groups (Wei et al., 2011; Consonni et al., 2012; Arana et al., 2015; Ciaramelli et al., 2018; Ciaramelli et al., 2019). SMA allowed not only the identification but also the simultaneous quantitation of all the metabolites over the detection limit (about 50 nM). Table 1 contains the corresponding quantification values.

**TABLE 1:** Metabolite quantification in GCE and RCE.

Metabolites	GCE		RCE	
	Mean	SD	Mean	SD
1,3-arabinofuranose unit	—	—	10.18	0.31
1,5-arabinofuranose unit	—	—	7.31	0.37
2-furylmethanol	—	—	0.59	0.08
5-CQA	128.5	2.61	42.54	1.64
5-hydroxymethyl-furfural	—	—	1.02	0.72
Acetate	0.67	0.06	4.12	0.18
Alanine	1.92	0.13	—	—
Caffeine	83.89	3.81	70.3	3.49
CGAs	349.59	6.68	148.57	13.96
Choline	16.31	0.15	2.2	0.04
Citrate	—	—	15.74	0.16
Formate	0.25	0.08	1.81	0.17
Lactate	12.62	0.48	6.71	0.68
Myoinositol	—	—	36.63	0.34
*N*-methylpyridinium	—	—	1.47	0.15
Nicotinic acid	—	—	0.27	0.06
Sucrose	204.01	2.79	—	—
Trigonelline	22.9	0.85	12.16	1.14

Results were expressed as µg/mg of extract and reported as mean and standard deviation (SD) of three-independent determinations (*n* = 3).

Melanoidin content in RCE was quantified by UV spectroscopy as 683.89 ± 33.99 μg/mg of RCE.

### Effect of Coffee Extracts and 5-CQA on THP-1-Derived Macrophages (TDM) Viability

**FIGURE 2:**
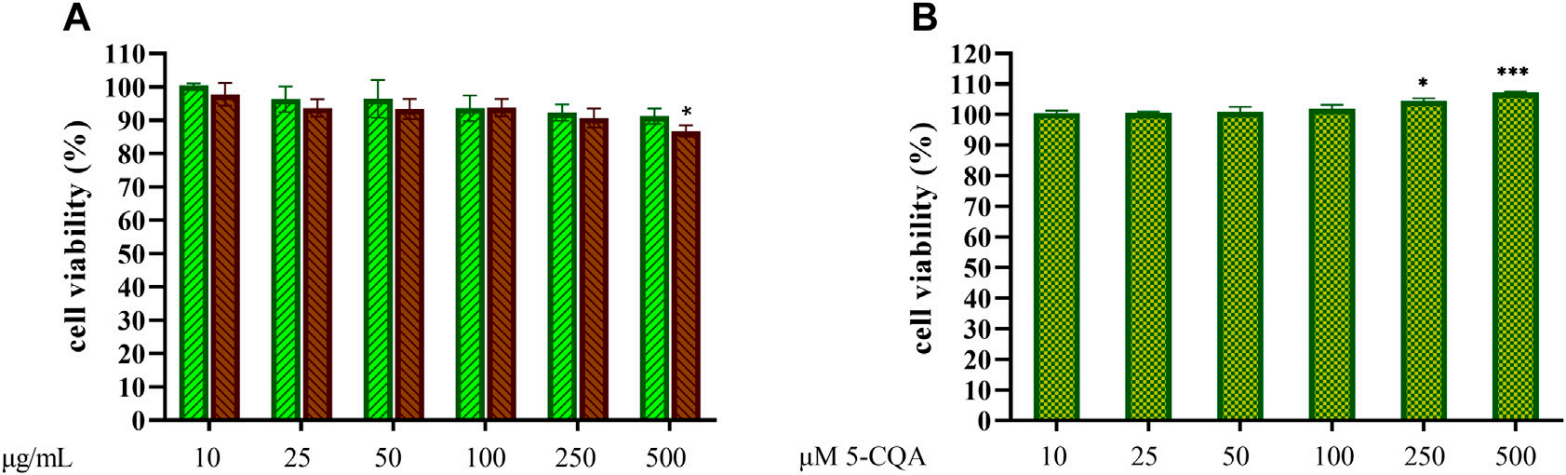
THP-1-derived macrophages viability. THP-1-derived macrophages (TDM) cell viability after treatment with increasing concentrations of GCE **(A**, green, down-up diagonal pattern**)** and RCE **(A**, brown, up-down diagonal pattern**)** and 5-CQA **(B**, square pattern**)** was evaluated by MTT assay (24 h). Data are represented as mean ± SEM of three independent experiments (*n* = 3). Results refer to untreated control (100%) (one-way ANOVA, *p < 0.05, **p < 0.01, ***p < 0.001, ****p < 0.0001).

The potential toxicity of coffee extracts on cells was firstly tested by treating THP-1-derived macrophages (TDM) with increasing concentrations of GCE and RCE (10–500 μg/ml) or 5-CQA (10–500 µM). After 24 h, cell supernatants were removed, and the remaining cell monolayers were immediately used to assess cytotoxicity *via* the MTT assay. The integrity of cell morphology before and after treatment was inspected by a light microscope. Treatment effect on cell viability was expressed by setting the percentage of non-treated cells at 100%. As shown in Figure 2A, viability of cells was generally unaffected by coffee extracts, except for RCE treatment of TDM at the highest concentration of 500 μg/ml. Therefore, for TDM, the highest concentration limit was set at 250 μg/ml in subsequent experiments. 5-CQA as well did not affect cell viability (Figure 2B).

### Effects of Coffee Extracts on Pro-inflammatory Cytokines Release in THP-1-Derived Macrophages

**FIGURE 3:**
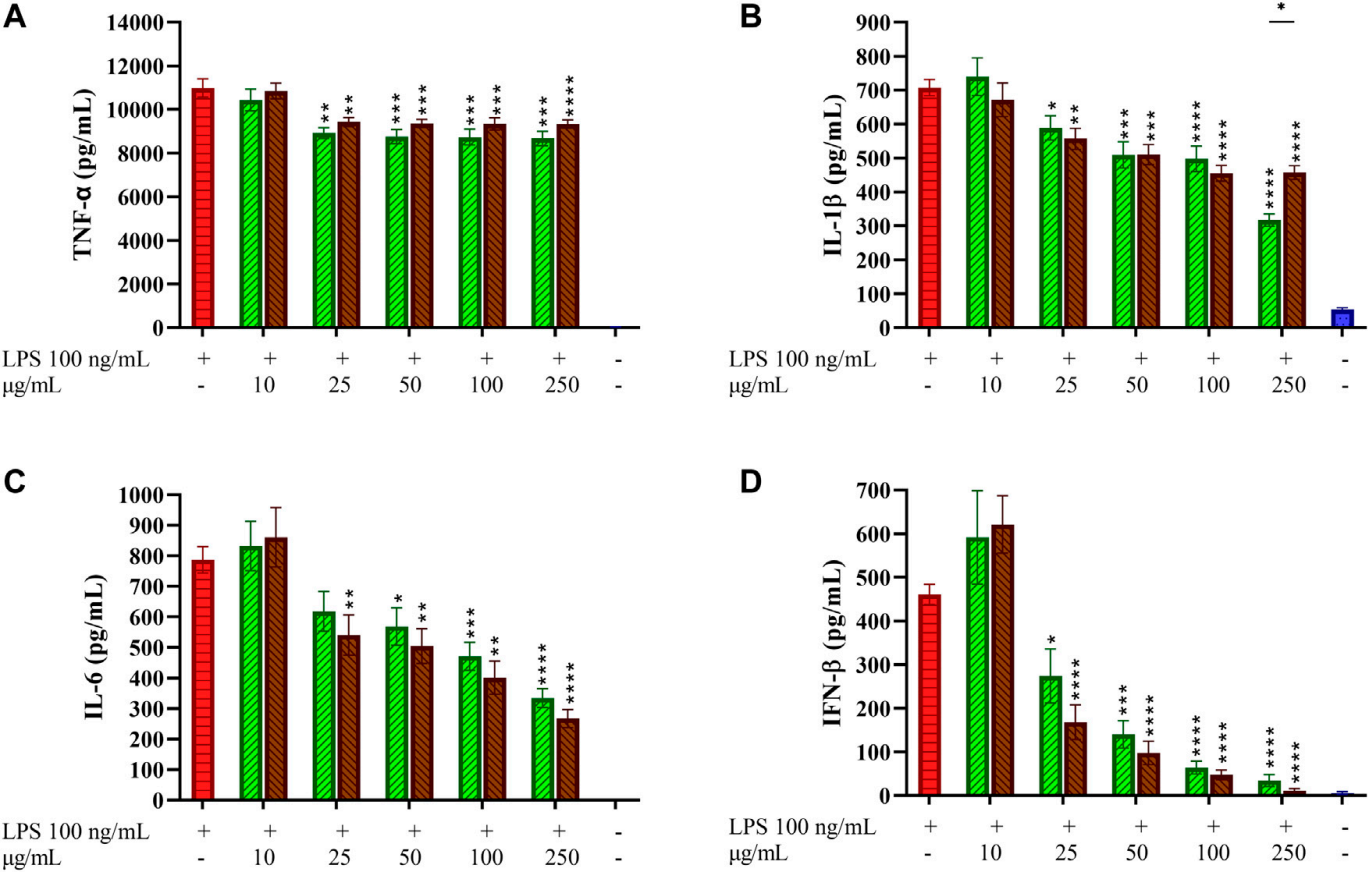
Pro-inflammatory cytokines profiling in LPS-stimulated THP-1-derived macrophages. THP-1-derived macrophages (TDM) cells were pre-treated with increasing concentrations of GCE (green, down-up diagonal pattern) and RCE (brown, up-down diagonal pattern) for 1 h and then challenged with 100 ng/ml LPS. TNF-α **(A)**, IL-1β **(B)**, IL-6 **(C)** and IFN-β **(D)** released in the medium after 24 h were quantified *via* ELISA. Data are represented as mean ± SEM of three independent experiments (*n* = 3). (one-way ANOVA, *p < 0.05, **p < 0.01, ***p < 0.001, ****p < 0.0001).

The effects of GCE and RCE on the production of the main inflammatory cytokines TNF-α, IL-6 and interleukin-1-β (IL-1β) produced downstream to the TLR4/MyD88 pathway was investigated in TDM. Cells have been treated with coffee extracts, then stimulated with LPS and cytokines were quantified in cell supernatants after 24 h from LPS stimulation. Pro-inflammatory cytokines levels of negative (non-treated) and positive (treated with LPS only) samples were compared with samples pre-treated with increasing concentrations of both GCE and RCE (10, 25, 50, 100, and 250 μg/ml) as depicted in Figure 3. Coffee extracts treatment of TDM resulted in a weak dose-dependent reduction of TNF-α release in response to LPS (Figure 3A). Contrarily, IL-1β and IL- 6 production was efficiently inhibited by coffee extracts. IL-1β release was more effectively reduced by CGE compared to RCE pre-treatment (Figure 3B). This different behavior was also confirmed by Pearson’s correlation analysis, which showed a significant linear correlation for GCE treatment but not for RCE treatment (Pearson *r* = −0.7391, *p* = 0.0932). IL-6 production in TDM was markedly reduced in a dose-dependent manner by both CGE and RCE pre-treatments (Figure 3C). TLR4 activation and signaling through the MyD88-independent TRAM/TRIF pathway leads to the activation of TBK1 and IRF-3 thus inducing the IFN-β gene transcription. As for other cytokines, IFN-β release was assessed by monitoring its concentration in cell supernatants after 3 h LPS stimulation. The IFN-β levels of negative (non-treated cells) and positive (cells treated with LPS only) samples were compared with samples pre-treated with increasing concentrations of GCE or RCE (10, 25, 50, 100, 250 or 500 μg/ml), as indicated in Figure 3D. A strong dose-dependent inhibition of IFN-β release was observed upon pre-treatment with both CGE and RCE.

### 5-CQA Inhibits IFN-β Release in THP-1-Derived Macrophages

**FIGURE 4:**
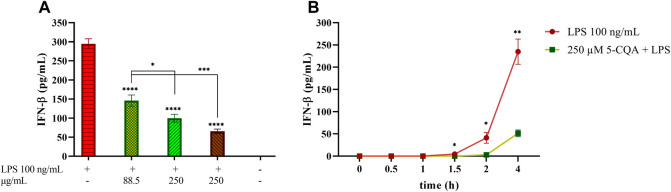
Effect of coffee extract and 5-CQA on THP-1-derived macrophages release of IFN-β upon LPS stimulation. **(A)** TDM cells were pre-treated with 88.5 μg/ml 5-CQA (dark green, square pattern), 250 μg/ml GCE (light green, down-up diagonal pattern) and 250 μg/ml RCE (brown, up-down diagonal pattern) for 1 h and then challenged with 100 ng/ml LPS. Supernatants were collected after 3 h. IFN-β released in the medium was quantified *via* ELISA. **(B)** THP-1-derived macrophages (TDM) cells were pre-treated with 250 μM 5-CQA (green squares) for 1 h and then challenged with 100 ng/ml LPS. TDM treated with LPS only are depicted in red circles. Supernatants were collected at different time points (0–4 h). IFN-β released in the medium was quantified *via* ELISA. Data are represented as mean ± SEM of three independent experiments (*n* = 3) (one-way ANOVA, *p < 0.05, **p < 0.01, ***p < 0.001, ****p < 0.0001).

The contribution of 5-CQA, one of the most abundant phytochemicals in coffee extracts, to this biological effect was then investigated. TDM cells were treated with 5-CQA (250 μM, corresponding to 88.5 μg/ml), GCE (250 μg/ml) and RCE (250 μg/ml) prior to be challenged with LPS (Figure 4A). The concentration of pure 5-CQA used in this experiment (88.5 μg/ml) reproduces the total concentration of chlorogenic acid present in 250 μg/ml GCE. ELISA quantification of IFN-β released in the medium showed the ability of 5-CQA to inhibit IFN-β release. IFN-β inhibition by 5-CQA was also investigated in time course experiments, by pre-treating cells with 5-CQA (1 h), challenging cells with LPS and then collecting supernatants at different time points (0–4 h). Figure 4B shows that LPS-induced IFN-β release starts between 1.5 and 2 h and it is higher at 4 h. Pre-treatment with 5-CQA dramatically decreases IFN-β release over time.

### Effect of Coffee Extracts and 5-CQA on NF-κB Activation in THP1-XBlue^™^ Cells

**FIGURE 5:**
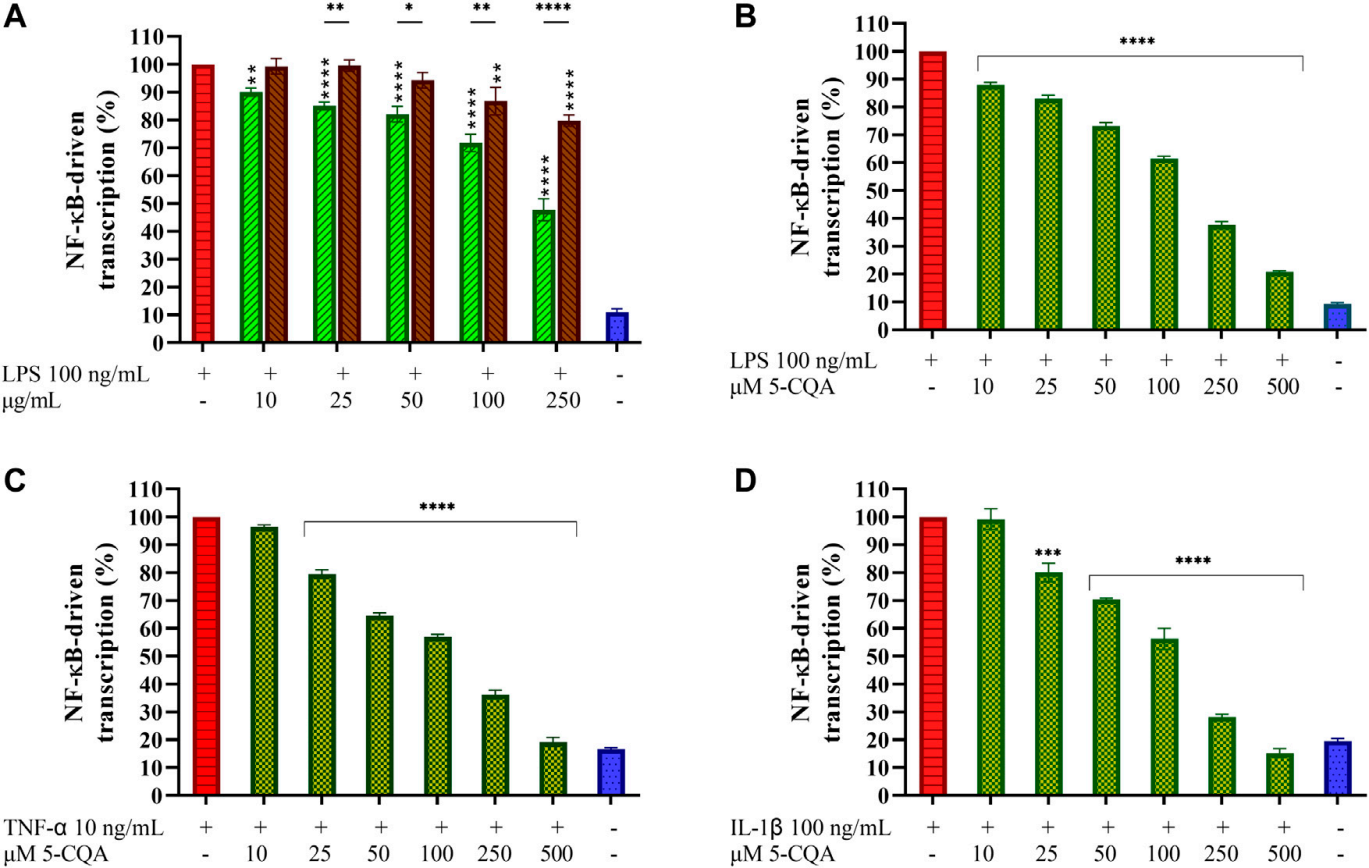
Effect of Coffee Extracts and 5-CQA on NF-κB-dependent transcription in THP1-XBlue^™^-derived macrophages. **(A)** THP-1-derived macrophages (TDM) cells treated with increasing concentrations of GCE (green, down-up diagonal pattern) and RCE (brown, up-down diagonal pattern) for 1 h and then challenged with 100 ng/ml LPS. **(B)** TDM cells pre-treated with increasing concentrations of 5-CQA (dark green, square pattern) for 1 h and then challenged with 100 ng/ml LPS. **(C)** TDM cells pre-treated with increasing concentrations of 5-CQA (dark green, square pattern) for 1 h and then challenged with 10 ng/ml TNF-α. **(D)** TDM cells pre-treated with increasing concentrations of 5-CQA (dark green, square pattern) for 1 h and then challenged with 100 ng/ml IL-1β. Activation of the NF-κB pathway was assessed by using THP1-XBlue^™^ cells as a reporter cell line and quantifying the activity of SEAP released in the medium after 18 h. Results refer to positive control (100%). Data are represented as mean ± SEM of three independent experiments (*n* = 3). Results are referred to untreated control (100%) (one-way ANOVA, *p < 0.05, **p < 0.01, ***p < 0.001, ****p < 0.0001).

LPS stimulation of cells induces NF-κB activation by phosphorylation and subsequent activation of downstream cytokines gene expression. To assess the effect of coffee extracts and 5-CQA on the LPS-induced NF-κB activation, a transcriptional reporter assay was used. In the absence of stimuli, NF-κB is associated with the inhibitory protein IκBα, which restrains translocation of the transcription factor from cytoplasm to the nucleus. LPS stimulation causes rapid IκBα degradation, allowing NF-κB activation by phosphorylation. The ability of GCE and RCE to counteract the activation of NF-κB occurring upon LPS-stimulation was assessed by using macrophages generated from THP1-XBlue^™^ cells, an engineered THP-1 cell line which expresses a reporter gene under the control of the NF-κB promoter. In these experimental conditions, LPS markedly increased the activation of NF-κB compared to the unstimulated control (Figure 5A). Both extracts inhibited NF-κB-driven transcription in a dose-dependent manner. GCE appears to be more active than RCE. Indeed, GCE pre-treatment results in a 50% inhibition at the maximum dose of 250 μg/ml, while with RCE pre-treatment 20% inhibition was observed at the same concentration. To better characterize the biological effects of 5-CQA observed on TDM we perform a transcriptional reporter assay employing THP1-XBlue^™^ cells, as we did for coffee extracts. After differentiation into macrophages, cells were treated with increasing concentrations of 5-CQA (10–500 μM) and then stimulated with 100 ng/ml LPS. Treatment with LPS results in a prominent release of SEAP as a consequence of NF-κB-driven transcription, while cells treated with 5-CQA only do not exhibit NF-κB activation (**data not shown**). Pre-treatment of cells with 5-CQA 1 h before the addition of LPS significantly inhibits NF-κB-driven transcription of SEAP in a dose-dependent manner (Figure 5B). Two pro-inflammatory stimuli different from LPS and capable of triggering the NF-κB pathway without targeting TLR4 were also used, namely the cytokines TNF-α and IL-1β. In both cases, 5-CQA pre-treatment was able to inhibit NF-κB-driven transcription of SEAP causes by both the stimulation with 10 ng/ml TNF-α (Figure 5C) and 100 ng/ml IL-1β (Figure 5D).

### Green and Roasted Coffee Extracts Inhibit p-IRF-3 Nuclear Translocation in THP-1-Derived Macrophages

**FIGURE 6:**
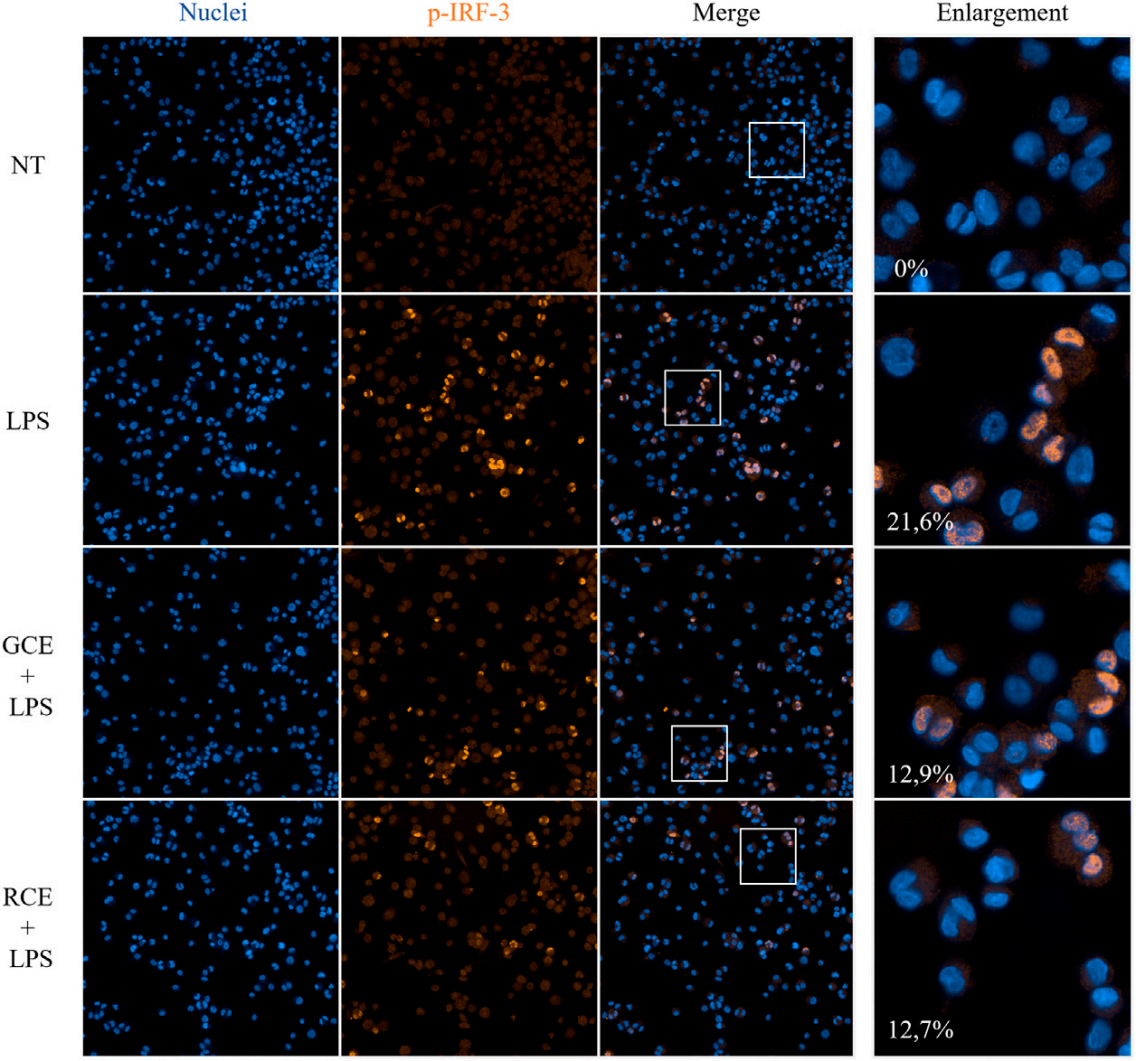
Immunofluorescence analysis of p-IRF-3 nuclear translocation. Phospho-IRF-3 localization in THP-1-derived macrophages (TDM) after 250 μg/ml GCE or RCE pre-treatment (1 h) and LPS stimulation (2 h). Images have been acquired using the Operetta CLS^™^ High-Content Analysis System and analyzed through Harmony 4.5 software with the following settings: original magnification ×20, water objective, confocal mode. Pictures are representative. Data represents the percentage of positive nuclei (nuclear fluorescence above a certain threshold) and are expressed as mean of different fields of view acquired (*n* = 9).

To investigate the subcellular events linked to the effects of GCE and RCE on inflammatory pathways and in particular on IFN-β production, we performed a confocal microscopy analysis by using an automated screening microscope, the Operetta CLS^™^ High-Content Analysis System. We investigated the effect of coffee extracts pre-treatment on nuclear translocation of the phosphorylated form of IRF-3 (Figure 6). The TLR4/TRIF pathway leads mainly to IRF-3 activation (Akira and Takeda 2004; Satoh and Akira 2016), and subsequent production of IFN-β. We therefore verified the activation of TLR4/TRIF pathway by monitoring the downstream activation of IRF-3. LPS stimulation of TDM cells triggered the phosphorylation of IRF-3 resulting in a fluorescence signal. Nuclei-located fluorescence signal in LPS-stimulated samples was higher than in non-treated samples. Treatment with RCE and GCE turned out to inhibit this event, resulting in a significant reduction of the nuclear translocation and, therefore, in the nuclei-associated fluorescence (Figure 6). This observation parallels with the finding of the strong inhibitory effect of RCE, and GCE, on IFN-β release.

### Green and Roasted Coffee Extracts Inhibit Interferon-β Release in CD14^+^ Monocyte-Derived Macrophages

**FIGURE 7:**
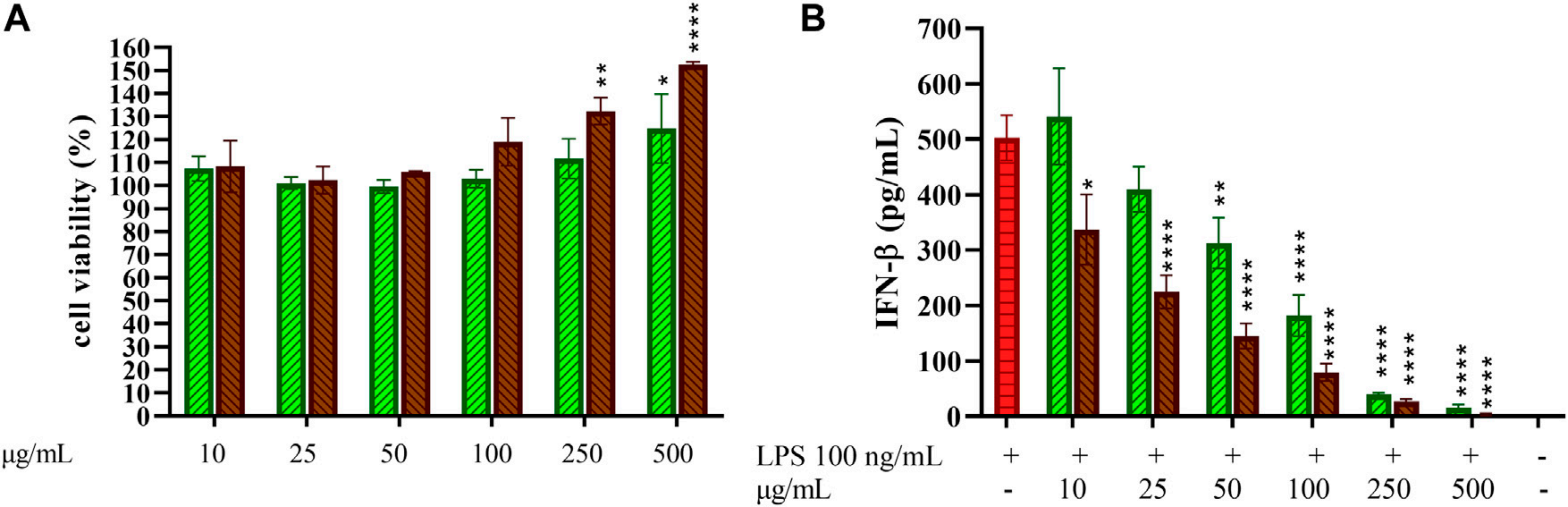
Effect of 5-CQA on CD14+ monocytes-derived macrophages cell viability and IFN-β release. **(A)** CD14^+^ monocytes-derived macrophages (MDM) cell viability after treatment with increasing concentrations of GCE (**A**, green, down-up diagonal pattern) and RCE (**A**, brown, up-down diagonal pattern) was evaluated by MTT assay (24 h). **(B)** MDM cells were pre-treated with increasing concentrations of GCE (light green, down-up diagonal pattern) and RCE (brown, up-down diagonal pattern) for 1 h and then challenged with 100 ng/ml LPS. Supernatants were collected after 3 h. IFN-β released in the medium was quantified *via* ELISA. Data are represented as mean ± SEM of three independent experiments (*n* = 3) (one-way ANOVA, *p < 0.05, **p < 0.01, ***p < 0.001, ****p < 0.0001).

Macrophage-like cells differentiated from purified CD14^+^ monocytes (MDM) were treated with increasing concentrations of GCE and RCE (10–500 μg/ml) to first assess toxicity of coffee extracts on primary cells. Cell viability after treatments is depicted in Figure 7A. None of the concentration used negatively affected cell viability, therefore the entire dose curve was maintained in subsequent experiments. Given the remarkable results obtained regarding the inhibition of IFN-β release in THP-1-derived macrophages, we were strongly encouraged to see if this effect should be confirmed on primary cells too. Thus, we treated MDM with GCE or RCE for 1 h and then we added LPS. As in previous experiments, we collected supernatants after 3 h and we measured the IFN-β released in the medium. As shown in Figure 7B, GCE and RCE are both able to decrease the amount of IFN-β released in a dose-dependent manner. These data are consistent with those found using TDM (Figure 3).

## Discussion

Bioactive plant secondary metabolites contained in significant amounts in food and beverages exhibit promising and still unexploited potential in the prevention of certain chronic inflammatory diseases that are characterized by the dysregulation of immune signaling pathways. Several studies showed the anti-inflammatory properties of coffee extracts in terms of inhibition of the main inflammatory mediators released upon LPS stimulation, both in murine cell lines as well as in mice. Nevertheless, very few data are available in literature regarding the immunomodulatory effects of coffee extracts and coffee-derived compounds on cells of human origin. As far as we know, this is the first investigation of coffee extracts effects on another inflammatory pathway occurring after LPS challenge, the TRIF-dependent cascade that leads to Type I interferon production.

First, this study reports a qualitative and quantitative characterization of the GCE and RCE molecular components by NMR. To summarize, the roasting process results in a reduction of total CGAs, and the formation of polymeric melanoidins together with other small molecules, such as *N*-methyl pyridinium, nicotinic acid, 5-hydroxy-methyl furfural and 2-furylmethanol. On the contrary some small molecular components such as trigonelline and choline are reduced during the roasting process. Among the main molecular components of both GCE and RCE there are the chemically heterogeneous CGAs, being 5-CQA the most abundant isomer. Therefore, alongside the study of the immunomodulatory effects of coffee extracts, we also investigated the biological effects of 5-CQA. Both GCE and RCE showed to be able to modulate inflammation in LPS-stimulated human TDM. Interestingly, the release of IL-1β and IL-6 was clearly inhibited by coffee extracts while TNF-α production was only slightly affected. These findings parallel with the results of a study on the effects of coffee charcoal extract (1–500 μg/ml) and chlorogenic acid isomers on TNF, IL-6, MCP-1 release from LPS-treated human THP-1 macrophages. Coffee charcoal extract showed concentration-dependent mild inhibitory effects towards TNF-α release and a more prominent inhibition of MCP-1 and IL-6 cytokines (Weber et al., 2019). The effects on TNF-α and IL-6 are quite similar to what we observed in the case of GCE and RCE. Another study on LPS-stimulated RAW 264.7 macrophage showed that the expression of mRNA for TNF-α and IL-6 was decreased in cells treated with coffee extracts.(Jung et al., 2017). Together, those data suggest that coffee extracts can modulate MyD88-dependent pro-inflammatory mediators.

We also investigated the putative modulatory effects of coffee extracts on the IFN-β secretion deriving from the TRIF-dependent TLR4 signaling. We report here for the first time that LPS-induced IFN-β release is inhibited by coffee extracts in human TDM. Inhibition of IFN-β was much more pronounced compared to the other cytokines examined: whether we observed a 50% inhibition of IL-6 and IL-1β release with extracts concentration of 250 μg/ml, the same IFN-β inhibition occurred with 25 μg/ml coffee extracts. To further investigate the mechanism of action of coffee extracts and confirm their inhibitory activity on the TRIF/IFN-β axis, time course confocal microscopy analysis was performed. Both extracts inhibited p-IRF-3 nuclear translocation, a key event within the TLR4 signaling that leads to IFN-β production, corroborating the inhibition of IFN-β release observed previously. On the way to confirm this novel biological effect of coffee extracts and the trustworthiness of our cell model, we reproduced the same experimental setting employing human primary cells *ex vivo*. We purified CD14^+^ monocytes from fresh human blood and differentiated them into macrophage-like cells (MDM). Interestingly, a robust dose-dependent inhibition of the IFN-β release was observed for both coffee extracts in primary human macrophages also, suggesting our model was reliable and opening translational perspectives. Chlorogenic acid is well-known for its anti-inflammatory effects. 5-CQA exerts anti-inflammatory activity by downregulating pro-inflammatory cytokines and interleukins. For instance, chlorogenic acid attenuated IL-1β, TNF-α, and IL-6 production in LPS-stimulated murine RAW 264.7 macrophages by effectively down regulating the NF-κB pathway (Hwang et al., 2014). Another cell-based study conducted on murine RAW 264.7 macrophages confirmed that CGA showed anti-inflammatory activity by suppressing LPS-induced COX-2 expression *via* attenuating the activation of NF-κB and JNK/AP-1 signaling pathways (Shan et al., 2009). Thus, we investigated if 5-CQA could contribute to the IFN-β modulation exerted by coffee extracts in human macrophages. Results showed the capability of 5-CQA to counteract IFN-β secretion, clearly indicating its involvement in the biological effect exerted by coffee extracts, even though treatment with whole extracts resulted in a higher inhibition. Time course experiments (0–4 h) had confirmed 5-CQA ability to modulate IFN-β as it is released upon LPS stimulation. Inappropriate activation of type I IFN can be detrimental to the host by promoting autoinflammatory responses and a break of immune tolerance, leading to autoimmunity. In fact, type I IFN activation is a key event in the pathogenesis of type I interferonopathies. Here, we report for the first time a remarkable effect of 5-CQA in inhibiting IFN-β release. A further characterization of the mechanism of action of 5-CQA pure component was performed involving the use of cell reporter assays as tools. Human macrophages obtained from the differentiation of THP1-XBlue^™^ cells showed the ability of extracts and 5-CQA to inhibit the NF-κB-dependent transcription that occurs upon stimulation with LPS. Moreover, cells pre-treated with pure 5-CQA and then challenged with inflammatory stimuli different from LPS, TNF-α and IL-1β, which recognition and initiation of the signaling is independent from TLR4, exhibited a diminished NF-κB activation, thus suggesting that the molecular targets of this compound might belong to the inflammatory cascade downstream the stimulus recognition and the 5-CQA mechanism of action is not membrane receptor-mediated. Since NF-κB is known to be involved in IFN-β expression, and LPS is known to induce both NF-κB and IRF-3, either the extracts and 5-CQA may inhibit IFN-β through diminished both IRF-3 and NF-κB translocation. However, further work is needed to mechanistically elucidate how extracts (and 5-CQA) cause the inhibition of IFN-β secretion.

Low bioavailability of their bioactive components can represent a limitation to the use of natural extracts as functional foods. The levels of CGAs in coffee brews range from 50 to 200 mg/100 ml, where on average 5-CQA accounting for the 41–48%. Based on the value of 100 mg CGAs/100 ml as the average of commonly reported contents in filtered coffee, the amounts of ingested CGAs per serving of 25–600 ml can be estimated in approximately 25–600 mg (Farah and dePaula Lima 2019). CGA isomers and di-caffeoylquinic acid isomers are highly absorbed and bioavailable for hours in plasma of green coffee consumers (Farah et al., 2008), as well as that of roasted coffee consumers (Monteiro et al., 2007). Concerning human studies, maximum plasma concentration (*C*_max_) of CGAs and metabolites vary with dose, individual, and with analytical methodology applied in the studies, ranging from nM to low µM levels(Farah and dePaula Lima 2019).

To the best of our knowledge, these results represent the first *in vitro* and *ex vivo* characterization of the IFN-β modulation by coffee extracts and the pure component 5-CQA in human macrophages (TDM and MDM). Our work provides the rationale to propose coffee as a relevant dietary source for bioactive immunomodulatory phytochemicals and considering 5-CQA as a lead compound for its biological activity which could be therapeutically useful both in intervention and prevention approaches. Collectively, our findings can be particularly relevant in the context of those disorders characterized by the constitutive overproduction of type I interferons.

## Data Availability

The original contributions presented in the study are included in the article, further inquiries can be directed to the corresponding author.
